# Response rate in the Study of Cardiovascular Risks in Adolescents – ERICA

**DOI:** 10.1590/S01518-8787.2016050006730

**Published:** 2016-02-02

**Authors:** Thiago Luiz Nogueira da Silva, Carlos Henrique Klein, Amanda de Moura Souza, Laura Augusta Barufaldi, Gabriela de Azevedo Abreu, Maria Cristina Caetano Kuschnir, Mauricio Teixeira Leite de Vasconcellos, Katia Vergetti Bloch

**Affiliations:** I Instituto de Estudos em Saúde Coletiva. Universidade Federal do Rio de Janeiro. Rio de Janeiro, RJ, Brasil; II Departamento de Epidemiologia. Escola Nacional de Saúde Pública Sérgio Arouca. Fundação Oswaldo Cruz. Rio de Janeiro, RJ, Brasil; III Departamento de Vigilância de Doenças e Agravos Não Transmissíveis e Promoção da Saúde. Secretaria de Vigilância em Saúde. Ministério da Saúde. Brasília, DF, Brasil; IV Programa de Pós-Graduação em Saúde Coletiva. Instituto de Medicina Social. Universidade do Estado do Rio de Janeiro. Rio de Janeiro, RJ,Brasil; V Núcleo de Estudos da Saúde do Adolescente. Faculdade de Ciência Médicas. Universidade do Estado do Rio de Janeiro. Rio de Janeiro, RJ, Brasil; VI Escola Nacional de Ciências Estatísticas. Fundação Instituto Brasileiro de Geografia e Estatística. Rio de Janeiro, RJ, Brasil

**Keywords:** Adolescent, Health Surveys, Data Collection, Response rate

## Abstract

**OBJECTIVE:**

To describe the response rate and characteristics of people who either took part or not in from the Study of Cardiovascular Risks in Adolescents (ERICA) , according to information subsets.

**METHODS:**

ERICA is a school-based, nation-wide investigation with a representative sample of 12 to 17-year-old adolescents attending public or private schools in municipalities with over 100,000 inhabitants in Brazil. Response rate of eligible subjects were calculated according to macro-regions, sex, age, and type of school (public or private). We also calculated the percentages of replacement schools in comparison with the ones originally selected as per the sample design, according to the types of schools in the macro-regions. The subjects and non-subjects were compared according to sex, age, and average body mass indices (kg/m^2^).

**RESULTS:**

We had 102,327 eligible adolescents enrolled in the groups drawn. The highest percentage of complete information was obtained for the subset of the questionnaire (72.9%). Complete information regarding anthropometric measurements and the ones from the questionnaire were obtained for 72.0% of the adolescents, and the combination of these data with the 24-hour dietary recall were obtained for 70.3% of the adolescents. Complete information from the questionnaire plus biochemical blood evaluation data were obtained for 52.5% of the morning session adolescents (selected for blood tests). The response percentage in private schools was higher than the one in public schools for most of the combination of information. The ratio of older and male adolescents non-participants was higher than the ratio among participants.

**CONCLUSIONS:**

The response rate for non-invasive procedures was high. The response rate for blood collection – an invasive procedure that requires a 12-hour fasting period and the informed consent form from legal guardians – was lower. The response rate observed in public schools was lower than in the private ones, and that may reflect lower school frequency of registered students.

## INTRODUCTION

Brazil’s health investigations allow discovering the distribution and magnitude of diseases as well as the social, economic, and environmental factors influencing them. They are therefore essential to plan and monitor health care interventions^7^. Changes in Brazil’s epidemiological pattern, characterized by the increased prevalence of chronic non-communicable diseases^11^, reaffirm the importance of conducting specific investigations to study these illnesses and their risk factors.

The Study of Cardiovascular Risks in Adolescents (ERICA) was developed to estimate the prevalence of metabolic syndrome and obesity in Brazilian adolescents and to outline the profile of risk factors for cardiovascular diseases, such as level of lipids, arterial blood pressure, inflammatory, and insulin resistance markers. Its database gathers several sets of information, a questionnaire, anthropometric measurements, dietary recall, and biochemical blood parameters, which serve to various purposes.

The response rate in epidemiological studies, regardless of study designs, has decreased throughout the years. Despite that, studies describing this response percentage and the characteristics of subjects and non-subjects are scarce^8^ ^,^ ^9^ ^,^ ^14^. The higher the response rate in a study, the lower the probability of results being affected by the compromising of internal validity due to self-selection biases^4^ ^,^ ^8^.

This study aimed to describe the response rate and characteristics of people who either took part or not in the ERICA, according to information subsets.

## METHODS

The ERICA is a school-based, countrywide investigation. Its sample was designed to be representative of 12 to 17-year-old adolescents attending morning or afternoon session in public or private schools, in municipalities with over 100,000 inhabitants in Brazil.

It is a complex sample with 32 strata composed of Brazilian capital states and the sets comprising municipalities with over 100,000 inhabitants in countryside areas of Brazilian macro-regions. In each stratum, the schools that had the most chances of being selected were the ones with the highest numbers of students enrolled in the seventh, eighth, and ninth grade of elementary school and in the first, second, and third grade of high school. The ones with the least chances of being selected were the ones located the farthest away from state capitals. In those schools, three combinations of grades and sessions (morning and afternoon) were selected. One group was selected in each combination, and all its students were invited to take part in the study. In total, 1,251 schools in 124 municipalities were selected, from a total of 273 municipalities with over 100,000 inhabitants. The study results are nationally representative of the 32 strata and the five Brazilian macro-regions. Details on the sampling design can be found in Vasconcellos et al.^15^

The principals of drawn schools were contacted, and when they refused to participate, we replaced the school by another one of the same stratum – the ones that had the best chances of being included were the nearest ones, with the same kind of administration (i.e., public or private), with the same urban or rural location, and with the same selection of grade and session combinations. The requirements from the research ethics committees for obtaining information varied from state to state. All patients signed informed consent forms before blood collection. However, for the remaining data (questionnaire, anthropometrics, measuring of arterial blood pressure, and dietary recall) only the students’ consent was required, except in four states (Goias, Bahia, Roraima, and Mato Grosso do Sul) and at public schools in Minas Gerais that required legal guardians to sign informed consent terms. The experiment protocol is described in detail in Bloch et al.^3^

We collected the data in a way to ensure privacy to each student, and the collection was conducted in the following order:

- Adolescent questionnaire: it comprised specific questions for each of the 11 thematic blocks (sociodemographic characteristics, work and employment, physical activity, eating behavior, smoking, alcohol consumption, reproductive health, oral health, self-reported morbidity, sleep duration, and common mental disorder). The questionnaire was self-filled out by personal digital assistant (PDA) and had around 105 questions (numbers of questions varied according to sex). The system through which the questionnaire was answered did not allow to skip questions – even if an adolescent reported not having a certain habit, they had to answer questions regarding it. This strategy aimed to assess the consistency of answers and to avoid that students could deny a habit to abstain from giving details about it later. Some questions include options such as: “I do not know/I do not remember/I do not want to answer”^3^.
- Anthropometric measurements (performed by trained observers): The adolescents’ weight was calculated (single measure) by a Líder^®^ electronic scale with capacity of 200 kilograms and variation of 50 grams; their height was obtained through the average of two sequential measurements of a portable, retractable stadiometer (Alturexata^®^), with resolution in millimeters and maximum range of 213 centimeters; their waist circumference was obtained through the average of two sequential measurements, using a fiberglass tape measure with resolution of 1 millimeter and length of 1.5 meters; and their arm circumference was obtained through a single measurement, by placing the measure around the arm at the midpoint between their acromion and their olecranon^3^.
- Arterial blood pressure and heartbeats: evaluated through three consecutive measurements by trained observers, with a three-minute interval between each measurement through a digital monitor (Omron 705-IT) validated for use in adolescents^12^. The measurements were taken from the adolescents’ right arms while they were sitting and their feet were on the floor, with proper cuffs for each arm size. Only the average of the three last measurements was used^3^.
- 24-hour dietary recall: food intake regarding the last 24 hours before the interview was registered by trained interviewers in laptops with softwares specifically developed for the task.
- Blood collection: blood samples were only collected from morning period students who had fasted for 12 hours. We measured the following items: total cholesterol, HDL-cholesterol, triglycerides, glycated hemoglobin, and insulin. These parameters were collected at the schools on dates exclusively scheduled for the purpose^3^.

The information regarding the number of students enrolled in the groups selected to take part in the study was obtained in the selected schools. Updated information was collected concerning the names and birth dates of students effectively attending the groups selected. Data on the frequency of registered students was obtained in the groups by respective teachers and principals.

The general collection strategy was coordinated by the central team of the project. However, each state had a local coordination that was in charge of all aspects involving logistics, recruitment and supervision of all supervisors trained by the central coordination, and also of all steps in the process to obtain the information, which was conducted at the schools by field researchers that had been hired and trained^3^.

The age and the sex of the students were also registered in the self-administered questionnaire. The information regarding birth date and age was also used to identify adolescents eligible for the study, as students younger than 12 and older than 17 years could be enrolled in the selected groups. The strategy of inviting all students of eligible ages intended on making the collection logistics easier, including the highest possible number of students of target ages (12 to 17 years old), and avoiding embarrassing students who were not in the target age range by excluding them from this step. Students in the eligible age ranges in grades below than the seventh grade were not included. Adolescents outside the age range between 12 and 17 years, pregnant ones, and physically or mentally-challenged adolescents (either temporarily or permanently disabilities that did not allow the adolescents to have their anthropometric measurements taken by the instruments in the research) were excluded from the analysis.

We included the adolescents in subsets according to the set of information obtained to enable the calculation of sample weights. The criteria to include or exclude study subjects who were eligible to be part of the groups with complete sets of information were:

- Questionnaire: included when all the questions in the 11 thematic blocks of the student questionnaire had been answered.
- Anthropometrics: included when at least the measurements regarding weight and height had been recorded.
- Arterial blood pressure: the invalid measurements were excluded (systolic arterial blood pressure less than or equal to diastolic arterial blood pressure).
- 24-hour dietary recall: the ones with energy consumption of less than 100 kcal were excluded.
- Blood: included adolescents with at least one biochemical test result among the all six.

Thus, adolescents might have filled out their questionnaire without having provided information on the dietary recall. Considering this, sample weights were calculated for each combination of these defined subsets, in a way to obtain unbiased estimates of the studied population. This resulted in 11 final subsets: 1) questionnaire; 2) questionnaire and anthropometrics; 3) questionnaire and dietary recall; 4) questionnaire, anthropometrics, and arterial pressure; 5) questionnaire, anthropometrics, and dietary recall; 6) questionnaire, anthropometrics, arterial blood pressure, and dietary recall; 7) questionnaire, and blood collection; 8) questionnaire, anthropometrics, and blood collection; 9) questionnaire, anthropometrics, arterial blood pressure, and blood collection; 10) questionnaire, anthropometrics, dietary recall, and blood collection; 11) questionnaire, anthropometrics, arterial blood pressure, dietary recall, and blood collection (Figure).

**Figure:**
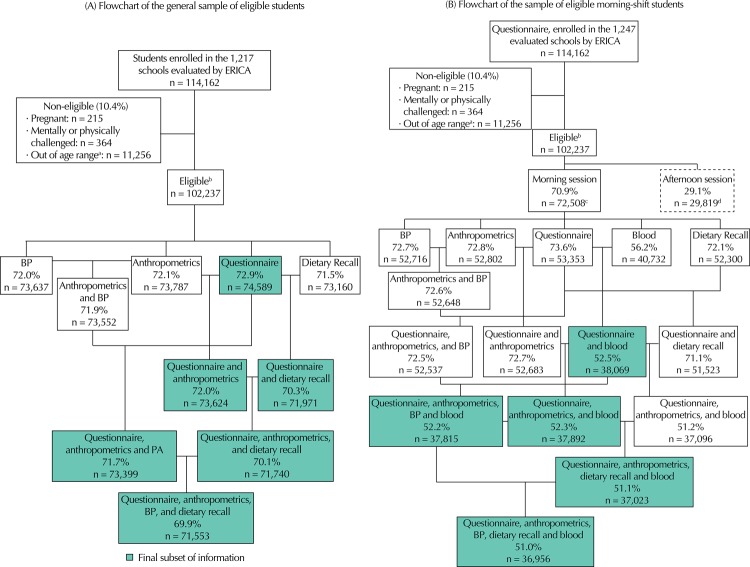
Flowchart of eligible adolescents and sample completeness regarding the information blocks and information subsets. ERICA, Brazil, 2013-2014.

ERICA: Study of Cardiovascular Risks in Adolescents; BP: arterial blood pressure

^a^ Younger than 12 and older than 17 years.

^b^ Among the 102,327 eligible students, 23.7% of them (24,284) did not answer any of the information blocks: questionnaire, anthropometrics, arterial blood pressure, and 24-hour dietary recall.

^c^ Among the 72,508 eligible morning shift students, 22.3% (16,131) did not answer any of the information blocks: questionnaire, anthropometrics, arterial blood pressure, and 24-hour dietary recall.

^d^ Among the 29,819 eligible afternoon shift students, 27.3% (8,153) did not answer any of the information blocks: questionnaire, anthropometrics, arterial blood pressure, and 24-hour dietary recall.

Effective response percentages were calculated; that is, the number of subjects with complete information according to the evaluated subset of information divided by the total number of eligible students (adolescents in the eligible age range enrolled in the groups), according to their macro-regions, sex, age (classified as 12 to 14, and 15 to 17 years), and types of school (public or private). We also calculated the percentage of replacement schools in relation to the ones selected in the original sample, according to types of schools in the macro-regions.

The subjects and non-subjects were compared according to their sex, age, and average body mass index (BMI; kg/m^2^). Information on sex and age of non-subjects was obtained from school files. BMI information was obtained from the students who only took part in the anthropometrics step and from the ones who gave information regarding weight and height but refused providing the remaining information of the study.

Non-subjects comprised absent students, students who missed school on the day scheduled for the data collection; partial losses were the ones who answered the questionnaire only partially, the ones with < 100 kcal in the dietary recall (24 hours), or the ones who did not have their weight or height measured; logistic losses were the ones who took part in any of the study steps, but did not have their information transferred to the central data server due to operational problems; refusal were the ones who, albeit being present, refused to take part in any of the steps.

## RESULTS

We started the collection in a public school in Rio de Janeiro, in the Southeast region, on March 18th, 2013, and finished it in a public school in Boa Vista, Roraima, North region, on November 10th, 2014.

Among the 102,327 students registered in the selected groups, 78,004 (76.2%) participated in at least one of the data collection steps of the study. In the total sample, the highest response rate was obtained for the information subset from the questionnaire, with percentages a little lower when anthropometric or dietary recall values were added (Figure, A). These percentages relate to information obtained from morning and afternoon session students. The response rate was smaller when blood tests were included, when only morning session students were considered (Figure, B). Generally speaking, these same relationships were observed according to macro-regions, sex, age ranges, and types of school (Table 1).

**Table 1:** Response percentage according to sex. age. and macro-regions. ERICA, Brazil, 2013-2014.

Sociodemographic variable	Number of students		Questionnaire (%)			
	
	Total	Morning session	A	B	C	D
Brazil	102,327	72,508	72.9	71.9	70.3	52.5
Sex						
Female	54,403	38,632	75.8	75.0	73.4	59.1
Male	47,924	33,876	69.6	68.5	66.9	45.0
Age range						
12-14	44,014	30,369	77.6	76.7	75.0	57.4
15-17	58,313	42,139	69.4	68.3	66.8	49.0
North	20,651	14,155	73.0	72.6	70.3	51.7
Sex						
Female	10,924	7,539	75.2	74.8	72.6	57.2
Male	9,727	6,616	70.5	70.1	67.7	45.5
Age range						
12-14	8,896	6,010	77.6	77.3	74.9	57.7
15-17	11,755	8,145	69.5	69.1	66.9	47.3
Northeast	31,754	22,211	73.0	72.0	70.4	53.2
Sex						
Female	17,063	11,914	75.4	74.6	72.9	59.9
Male	14,691	10,297	70.2	68.9	67.4	45.5
Age range						
12-14	13,572	9,734	77.4	76.6	74.9	57.1
15-17	18,182	12,477	69.6	68.6	66.9	50.2
Midwest	14,238	10,281	68.3	67.5	66.0	53.6
Sex						
Female	7,666	5,513	73.4	72.7	71.1	61.2
Male	6,572	4,768	62.3	61.3	60.0	44.9
Age range						
12-14	6,142	4,336	73.5	72.6	71.6	60.7
15-17	8,096	5,945	64.4	63.5	61.8	48.5
Southeast	23,911	17,506	71.4	70.4	69.2	49.4
Sex						
Female	12,640	9,337	74.8	74.1	72.8	56.5
Male	11,271	8,169	67.6	66.4	65.2	41.3
Age range						
12-14	10,260	6,907	77.1	76.3	74.8	53.8
15-17	13,651	10,599	67.2	66.0	65.0	46.6
South	11,773	8,355	81.0	79.2	77.8	56.9
Sex						
Female	6,110	4,329	82.9	81.2	80.0	63.0
Male	5,663	4,026	79.0	77.0	75.5	50.4
Age range						
12-14	5,144	3,382	83.7	82.1	80.2	60.9
15-17	6,629	4,973	79.0	76.9	75.9	54.2

A: Isolated questionnaire; B: + anthropometrics; C: + dietary recall; D: + blood parameters (Morning session).

The highest response percentages of the information subsets that include an isolated questionnaire and anthropometrics or dietary information were obtained in the South region, especially in the oldest age range, from 15 to 17 years, for both sex (Table 1). For the combination of questionnaire and blood information, the highest response rate took place with the youngest, 12 to 14-year-old, female students in the Midwest region (Table 2).

**Table 2:** Coverage of information according to macro-regions, sex, and age. ERICA, Brazil, 2013-2014.

Sociodemographic variable	Number of students		Questionnaire (%)			
	
	Total	Morning session	A	B	C	D
Brazil						
Sex						
Female	23,303	16,094	80.3	79.6	78.0	64.0
Male	31,100	22,538	72.4	71.5	69.9	55.6
Age range						
12-14	20,711	14,275	74.5	73.5	71.7	50.0
15-17	27,213	19,601	65.9	64.7	63.2	41.4
North						
Sex						
Female	4,722	3,196	79.2	78.9	76.8	62.4
Male	6,202	4,343	72.1	71.7	69.5	53.3
Age range						
12-14	4,174	2,814	75.9	75.6	72.8	52.4
15-17	5,553	3,802	66.5	66.1	63.9	40.5
Northeast						
Sex						
Female	7,290	5,217	80.3	79.7	77.7	64.5
Male	9,773	6,697	71.7	70.9	69.3	56.3
Age range						
12-14	6,282	4,517	74.1	72.9	71.7	48.6
15-17	8,409	5,780	67.2	65.9	64.2	43.1
Midwest						
Sex						
Female	3,244	2,291	78.5	77.8	77.0	68.0
Male	4,422	3,222	69.7	69.0	66.8	56.4
Age range						
12-14	2,898	2,045	67.8	66.8	65.6	52.5
15-17	3,674	2,723	58.0	57.0	55.7	39.1
Southeast						
Sex						
Female	5,419	3,653	79.8	79.2	77.7	60.4
Male	7,221	5,684	71.2	70.2	69.1	54.0
Age range						
12-14	4,841	3,254	74.1	73.0	71.5	46.4
15-17	6,430	4,915	62.7	61.4	60.4	38.0
South						
Sex						
Female	2,628	1,737	85.5	84.0	82.8	67.5
Male	3,482	2,592	81.0	79.0	77.8	60.1
Age range						
12-14	2,516	1,645	81.7	80.1	77.6	54.0
15-17	3,147	2,381	76.9	74.5	73.8	47.9

A: Isolated questionnaire; B: + anthropometrics; C: + dietary recall; D: + blood parameters (Morning session).

For most combinations, the response rate in the study was higher in private schools than in public ones. However, in the South region the differences were smaller and the response rate of 12 to 14-year-old adolescents was higher in the public schools for all combinations of information subsets; regarding the subset with blood information, the higher response rate in the public schools was observed for both age ranges and sex (Table 3).

**Table 3:** Response percentage according to sex, age, macro-regions, and types of school. ERICA, Brazil, 2013-2014.

Sociodemographic variable	Public schools						Private schools					
	
	Number of students		Questionnaire (%)				Number of students		Questionnaire (%)			
			
	Total	Morning session	A	B	C	D	Total	Morning session	A	B	C	D
Brazil	81,970	54,361	71.6	70.7	68.9	51.8	20,357	18,147	78.0	76.9	76.0	54.6
Sex												
Female	43,691	29,024	74.6	73.9	72.0	58.6	10,712	9,608	80.5	79.4	78.8	60.5
Male	38,279	25,337	68.2	67.1	65.4	44.0	9,645	8,539	75.3	74.1	73.0	47.9
Age range												
12-14	34,312	22,031	76.6	75.8	73.9	56.8	9,702	8,338	81.0	80.1	79.0	59.0
15-17	47,658	32,330	68.0	67.1	65.3	48.4	10,655	9,809	75.3	73.9	73.3	50.8
North	18,001	11,657	72.8	72.5	70.0	51.6	2,650	2,498	74.2	73.2	72.5	52.5
Sex												
Female	9,579	6,263	74.9	74.7	72.2	57.0	1,345	1,276	77.0	75.8	75.7	57.7
Male	8,422	5,394	70.4	70.1	67.5	45.2	1,305	1,222	71.4	70.6	69.2	47.1
Age range												
12-14	7,927	5,185	77.4	77.2	74.5	57.7	969	825	79.5	78.7	78.4	57.9
15-17	10,074	6,472	69.2	68.9	66.5	46.7	1,681	1,673	71.2	70.1	69.1	49.9
Northeast	23,704	14,741	71.0	70.1	68.1	52.2	8,050	7,470	78.6	77.6	76.8	55.3
Sex												
Female	12,774	7,913	73.3	72.6	70.6	59.1	4,289	4,001	81.5	80.6	79.6	61.3
Male	10,930	6,828	68.4	67.1	65.3	44.1	3,761	3,469	75.3	74.2	73.7	48.3
Age range												
12-14	9,199	5,757	75.9	75.0	73.0	56.3	4,373	3,977	80.7	79.8	79.0	58.3
15-17	14,505	8,984	68.0	67.0	65.1	49.6	3,677	3,493	76.0	75.0	74.2	51.8
Midwest	11,194	7,474	66.8	65.9	64.2	52.2	3,044	2,807	73.8	73.2	72.6	57.5
Sex												
Female	6,022	3,993	72.4	71.6	69.8	60.5	1,644	1,520	77.2	76.7	76.2	63.1
Male	5,172	3,481	60.3	59.2	57.8	42.6	1,400	1,287	69.8	69.1	68.4	50.9
Age range												
12-14	4,640	2,965	71.8	70.8	69.7	58.8	1,502	1,371	78.6	78.2	77.5	64.8
15-17	6,554	4,509	63.3	62.4	60.3	47.8	1,542	1,436	69.1	68.3	67.8	50.6
Southeast	19,780	14,042	69.7	68.7	67.3	48.5	4,131	3,464	79.8	78.6	78.2	53.3
Sex												
Female	10,451	7,499	73.4	72.7	71.2	55.6	2,189	1,838	81.6	80.5	80.4	60.2
Male	9,329	6,543	65.5	64.3	63.0	40.3	1,942	1,626	77.8	76.5	75.7	45.5
Age range												
12-14	8,445	5,445	75.6	74.8	73.1	52.4	1,815	1,462	84.0	83.0	82.8	59.0
15-17	11,335	8,597	65.3	64.2	63.1	46.0	2,316	2,002	76.6	75.1	74.7	49.2
South	9,291	6,447	80.7	78.9	77.8	58.3	2,482	1,908	82.4	80.2	77.8	52.4
Sex												
Female	4,865	3,356	82.9	81.2	80.1	64.8	1,245	973	83.1	81.0	79.5	56.9
Male	4,426	3,091	78.3	76.3	75.3	51.2	1,237	935	81.7	79.4	76.0	47.7
Age range												
12-14	4,101	2,679	84.0	82.5	81.5	62.9	1,043	703	82.3	80.5	75.5	53.5
15-17	5,190	3,768	78.1	76.0	74.9	55.0	1,439	1,205	82.5	79.9	79.4	51.8

A: Isolated questionnaire; B: + anthropometrics; C: + dietary recall; D: + blood parameters (Morning session).

Out of the 1,251 selected schools in the original sample, four were lost: three in Sao Paulo and one in Amapa, all private schools with no replacements. In the end, 139 (11.1%) of the schools were replaced. Among the 1,012 public schools and the 235 private schools, these replacements took place in 6.0% and 32.2% of the cases, respectively. In all regions, the replacements were relatively more frequent in private schools. North region was found to have the smallest relative frequency of substitutions in private schools. However, the South region was found to have the extremes; that is, the smallest percentage of substitutions in public schools (3.8%) and the highest percentage in the private ones (48.4%). The questionnaire covered 71.7% of the public schools that were not replaced and 70.7% of the replaced ones, whereas in private schools, the corresponding percentages were 78.4% and 77.0% (Table 4).

**Table 4:** Response percentage of questionnaires of the originally-selected or replacement schools according to macro-regions. ERICA, Brazil, 2013-2014.

Macro-region	Public schools				Private schools			
	
	Original selection		Replacement schools		Original selection		Replacement schools	
			
	n	%	n	%	n	%	n	%
Brazil	77,459	71.7	4511	70.7	14,400	78.4	5,957	77.0
North	17,028	72.8	973	72.8	2,334	74.1	316	75.3
Northeast	22,336	70.9	1368	73.0	6,126	79.7	1,924	74.9
Midwest	10,773	66.9	421	64.8	2,246	76.3	798	66.8
Southeast	18,267	69.7	1513	69.3	2,372	78.5	1,759	81.6
South	9,055	81.0	236	68.2	1,322	83.7	1,160	80.9

Regarding the subset of information from the questionnaire and anthropometric data, among the 28,713 eligible non-subjects, most of them (77.2%) were absent on the day the information was collected in both steps, 5,272 (19.0%) of them were present and refused to participate, 109 (0.4%) of them were logistic losses, and 1,154 (4.0%) were partial losses (either had their weight and/or height measured, but have not answered the questionnaire; or only answered the questionnaire). BMI data were collected from 2,374 non-subject adolescents, of whom 2,214 (93.2%) were refusals and reported weights and heights, and 160 (6.8%) were partial losses and had their weight and height measured, despite not answering the questionnaire (Table 5).

**Table 5:** Percentages and body mass indices (BMI) of subjects and non-subjects. according to sex and age range as per macro-regions, in ERICA’s sample of eligible students. Brazil, 2013-2014.

Sociodemographic variable	Subjects^a^				Non-subjects					
	
	n	%	BMI	95%CI	n	%	n^b^	%	BMI	95%CI
Brazil	73,624	-	21.2	21.2-21.2	28,703	-	2,374	-	21.4	21.3-21.6
Sex										
Female	40,803	55.4	21.3	21.3-21.4	13,600	47.4	945	39.8	21.4	21.2-21.7
Male	32,821	44.6	21.0	21.0-21.0	15,103	52.6	1,429	60.2	21.4	21.2-21.7
Age range										
12-14	33,778	45.9	20.5	20.5-20.6	10,236	35.7	1,085	45.7	20.8	20.5-21.0
15-17	39,846	54.1	21.7	21.7-21.8	18,467	64.3	1,289	54.3	22.0	21.8-22.2
North	14,996	-	20.9	20.9-21.0	5,655	-	245	-	21.6	21.1-22.1
Sex										
Female	8,174	54.5	21.0	20.9-21.1	2,750	48.6	97.0	39.6	21.1	20.4-21.8
Male	6,822	45.5	20.8	20.7-20.9	2,905	51.4	148	60.4	21.9	21.2-22.6
Age range										
12-14	6,879	45.9	20.2	20.1-20.3	2,017	35.7	114	46.5	21.0	20.2-21.7
15-17	8,117	54.1	21.6	21.5-21.6	3,638	64.3	131	53.5	22.1	21.4-22.8
Northeast	22,864	-	21.1	21.0-21.1	8,890	-	244	-	21.8	21.2-22.4
Sex										
Female	12,737	55.7	21.2	21.1-21.2	4,326	48.7	98	40.2	22.2	21.1-23.2
Male	10,127	44.3	20.9	20.8-21.0	4,564	51.3	146	59.8	21.5	20.8-22.2
Age range										
12-14	10,390	45.4	20.5	20.4-20.6	3,182	35.8	97	39.8	21.5	20.4-22.5
15-17	12,474	54.6	21.5	21.5-21.6	5,708	64.2	147	60.2	22.0	21.3-22.7
Midwest	9,604	-	21.1	21.0-21.2	4,634	-	718	-	20.8	20.5-21.0
Sex										
Female	5,573	58.0	21.2	21.1-21.3	2,093	45.2	304	42.3	20.8	20.4-21.2
Male	4,031	42.0	20.9	20.8-21.0	2,541	54.8	414	57.7	20.8	20.4-21.1
Age range										
12-14	4,459	46.4	20.4	20.3-20.5	1,683	36.3	367	51.1	20.3	19.9-20.6
15-17	5,145	53.6	21.7	21.6-21.8	2,951	63.7	351	48.9	21.3	20.9-21.7
Southeast	16,841	-	21.3	21.3-21.4	7,070	-	602	-	21.6	21.2-21.9
Sex										
Female	9,360	55.6	21.6	21.5-21.7	3,280	46.4	225	37.4	21.9	21.2-22.6
Male	7,481	44.4	21.0	20.9-21.1	3,790	53.6	377	62.6	21.4	21.0-21.8
Age range										
12-14	7,827	46.5	20.7	20.6-20.8	2,433	34.4	266	44.2	20.8	20.3-21.4
15-17	9,014	53.5	21.9	21.9-22.0	4,637	65.6	336	55.8	22.2	21.7-22.7
South	9,319	-	21.8	21.7-21.8	2,454	-	565	-	22.0	21.6-22.3
Sex										
Female	4,959	53.2	21.9	21.3-22.0	1,151	46.9	221	39.1	21.7	21.3-22.2
Male	4,360	46.8	21.6	21.4-21.7	1,303	53.1	344	60.9	22.1	21.7-22.6
Age range										
12-14	4,223	45.3	21.1	21.0-21.2	921	37.5	241	42.7	21.2	20.7-21.7
15-17	5,096	54.7	22.3	22.2-22.4	1,533	62.5	324	57.3	22.5	22.1-22.9

^a^ Regarding the information subset from eligible students who filled out questionnaires and had anthropometric measurements taken.

^b^ Number of individuals with self-reported anthropometric information among the ones who refused to participated in the study (n = 2,214) or who took part in the anthropometric step but did not answer or managed to fill out the questionnaire (n = 160).

The ratio of male and those adolescents with 15 to 17 years old were higher among non-subjects as compared to the ERICA’s subjects. This setting was similar in all macro-regions in Brazil. In the total sample and in the macro-regions, the average BMI was a little higher among non-subjects (Table 5).

## DISCUSSION

Even though the large sample size of ERICA and the careful planning and execution of the study^3^ reinforce the significance of its findings, it is important to understand the dimension and the characteristics of the participation in the study considering possible biases that affect its internal quality.

Around 78,000 eligible adolescents had some information collected in the study, and we found variations in the response rate according to the information subsets. The highest response rate was found for the information subset collected in the self-administered questionnaire and the lowest one for the blood subset. Moreover, some differences among regions and regarding types of school were observed: the public schools had lower response percentages as compared to the private ones, with the exception of the South region, especially regarding blood collection.

The assessment of participation and non-participation in epidemiological studies and the detailed description of such lack of response is not often found in the literature^4^ ^,^ ^8^. It is very important that non-answers are adequately reported so that results can be interpreted properly and that strategies aiming to reduce non-answers may be developed^4^ ^,^ ^10^.

Morton et al.^8^ selected 355 epidemiological studies published between January 2003 and April 2003 in 10 international periodicals, and found 86 cross-sectional studies, of which only 51.0% reported participation data varying between 28.0% and 100%.

Country-wide population-based studies about adolescents are scarce^1^ ^,^ ^5^ ^,^ ^6^ ^,^ ^13^. *Pesquisa Nacional de Saúde do Escolar* (PeNSE – Brazil’s National Student Health Survey), (conducted with students in the ninth grade of elementary school^5^, found a response percentage of 83.0%, only considering the information subset from the questionnaire. The difference between the response percentages of ERICA and PeNSE may be partly accounted for the fact that the sample of PeNSE included 13 to 15-year-old adolescents, who were younger than the ones from ERICA. In ERICA, coverage of all information subsets was higher among 12 to 14-year-old adolescents, and the questionnaire subset had a 76.2% coverage for this age range. Another difference between the studies is ERICA’s requirement of informed consent terms for some states, in which we found lower coverage percentages. Midwest and South regions have the lowest and the highest coverage percentages, respectively, in both investigations.

As compared to international studies such as the Healthy Lifestyle in Europe by Nutrition in Adolescence (HELENA)^2^, a school study that was conducted in 10 European cities with 12 to 17-year-old adolescents, the coverage of ERICA was superior (67.0% *versus* 73.0%). In HELENA, in the subsample of adolescents who had their blood collected, the coverage for these information was superior to the one found in ERICA (68,0% *versus* 52,0%).

The response rate differences in the information subsets may be explained by the degree of logistic complexity required to obtain information. The need of informed consent terms signed for non-invasive procedures may have influenced the losses, including information subsets other than the blood one. Informed consent terms were not required in the South of the country; in the Southeast region, Minas Gerais Education Office was the only one to require them; in the remaining macro-regions, the only states that required them were Roraima in the North region, Bahia in the Northeast region, and Goias and Mato Grosso do Sul in the Midwest region. In the last four states, the coverage of information in the questionnaire was less than 60.0%. That may reflect the impact from the mandatory informed consent terms, as the other two states in the Midwest region, in which consent terms were not required had response percentages in the questionnaires above 80.0%.

The differences observed among the response percentages according to the types of school seemed to have no relation to the higher ratio of replacements in private schools, as the response percentages among the original schools in the sample and the replacement ones were similar regardless of their type of administration. It is possible to hypothesize that the coverage differences between public and private schools are related to absenteeism. In PeNSE 2012, among the public school students, 28.8% reported having missed classes without permission from their parents in the previous 30 days, whereas in private schools, this percentage was 14.4%. Other possible explanations: public schools are more frequently located in dangerous areas and the higher probability for those students having to work, which may increase absenteeism. Regarding that last hypothesis, in the total sample of eligible students, 6.2% from public schools had paying jobs, as compared to only 2.8% in private schools (missing data). The estimate for prevalence of public school students who reported working may be underestimated, as it was only obtained from the ones who were present on the collection day and answered the questionnaires.

The adolescents who did not participate in ERICA were mostly males and 15 to 17 years old. Moreover, they had BMI values slightly superior to the adolescents of this study. However, BMI data were only estimated in 8.5% of non-subjects, and this estimate may not be representative of the total non-subjects. In ERICA, we established analysis procedures that, besides considering complex sampling, used calibrated weights that adjusted estimates of prevalence according to the distribution of age and sex in each sample stratum, which was estimated with information obtained from population-based censuses^15^. Furthermore, these specific estimates consider that the subjects of ERICA represent those who did not participate. The possible accuracy losses in the estimates are more related to the level of clustering, due to the option of including all students from the selected groups than to the total number of observations, which was close to the expected one.

The ERICA adopted some strategies to foster student participation, such as: preparation of material with information on the study made by a famous children’s cartoonist, to attract the attention of the youngsters – it included a brochure, a poster, and the study slogan; distribution of food kits for the ones who fasted before the blood collection; and publishing of the study in a website (www.erica.ufrj.br), with a communication channel (contact us) and broadcasting of it on television and on local press media at the beginning of the collection in each state. These strategies increased the cost of the study, but are fundamental to reduce lack of participation in epidemiological studies. Similarly, strategies to reach non-subjects and obtain some information about them to understand their resistance to taking part are important and require great logistic effort that is hard and costly to implement in a large population-based study^4^.

The ERICA is the first nationwide, school-based investigation that obtained comprehensive information on nutritional state, arterial blood pressure, and biochemical blood parameters of adolescents. The non-answer ratio – lower than the third of the number of subjects – will hardly cause an impact in the estimates of the study under the characteristics described. This study provides useful information for future investigations in adolescents, which may improve strategies to collect information to reduce the lack of response.
