# Supplementary material for: Response rate in the Study of Cardiovascular Risks in Adolescents – ERICA
Source: Rev Saude Publica. 2016 Feb 2;50(Suppl 1):3s. doi: 10.1590/S01518-8787.2016050006730 (PMC4767034; doi:10.1590/S01518-8787.2016050006730)
Supplement: Translation

# Translation: Participação no Estudo de Riscos Cardiovasculares em Adolescentes - ERICA

**Authors:** Thiago Luiz Nogueira da Silva, Carlos Henrique Klein, Amanda de Moura Souza, Laura Augusta Barufaldi, Gabriela de Azevedo Abreu, Maria Cristina Caetano Kuschnir, Mauricio Teixeira Leite de Vasconcellos, Katia Vergetti Bloch

**Affiliations:** I Instituto de Estudos em Saúde Coletiva. Universidade Federal do Rio de Janeiro. Rio de Janeiro, RJ, Brasil; II Departamento de Epidemiologia. Escola Nacional de Saúde Pública Sérgio Arouca. Fundação Oswaldo Cruz. Rio de Janeiro, RJ, Brasil; III Departamento de Vigilância de Doenças e Agravos Não Transmissíveis e Promoção da Saúde. Secretaria de Vigilância em Saúde. Ministério da Saúde. Brasília, DF, Brasil; IV Programa de Pós-Graduação em Saúde Coletiva. Instituto de Medicina Social. Universidade do Estado do Rio de Janeiro. Rio de Janeiro, RJ, Brasil; V Núcleo de Estudos da Saúde do Adolescente. Faculdade de Ciência Médicas. Universidade do Estado do Rio de Janeiro. Rio de Janeiro, RJ, Brasil; VI Escola Nacional de Ciências Estatísticas. Fundação Instituto Brasileiro de Geografia e Estatística. Rio de Janeiro, RJ, Brasil

**Keywords:** Adolescente, Inquéritos Epidemiológicos, Coleta de Dados, Taxa de Resposta

## Abstract

**OBJETIVO:**

Descrever o percentual de resposta e características de participantes e não-participantes no Estudo de Riscos Cardiovasculares em Adolescentes (ERICA) segundo subconjuntos de informações.

**MÉTODOS:**

O ERICA é um inquérito de base escolar de abrangência nacional com amostra representativa de adolescentes, de 12 a 17 anos de idade, que estudam em escolas públicas ou privadas de municípios com mais de 100 mil habitantes de todo o Brasil. Foram calculadas frequências de participação (de elegíveis) por macrorregiões, sexo, idade, e tipo de escola (pública ou privada). Foram calculados também os percentuais de escolas substitutas das selecionadas na origem do desenho amostral, de acordo com o tipo de escola nas macrorregiões. Os participantes e não-participantes foram comparados segundo sexo, idade e média de índice de massa corporal (kg/m^2^).

**RESULTADOS:**

Estavam cadastrados nas turmas sorteadas 102.327 adolescentes elegíveis. O maior percentual de informações completas foi obtido para o subconjunto do questionário (72,9%). Informações completas das medidas antropométricas e do questionário foram obtidas em 72,0% dos adolescentes, e a combinação dessas informações com recordatório alimentar de 24h, em 70,3%. Informações completas do questionário mais avaliação bioquímica do sangue foram obtidas em 52,5% dos adolescentes do turno da manhã (elegíveis para exame de sangue). O percentual de resposta nas escolas privadas foi maior do que a das escolas públicas para a maioria das combinações de informações. A proporção de adolescentes não participantes do sexo masculino e mais velhos foi maior do que entre os participantes.

**CONCLUSÕES:**

O percentual de resposta para procedimentos não invasivos foi elevado. Para a coleta de sangue, procedimento invasivo, com necessidade de jejum de 12 horas e de consentimento escrito dos responsáveis, a participação foi menor. O percentual de participação observado nas escolas públicas foi menor do que nas particulares, podendo refletir menor frequência escolar dos alunos cadastrados.

## INTRODUÇÃO

Os inquéritos nacionais de saúde permitem conhecer a distribuição e magnitude das doenças, bem como de seus condicionantes sociais, econômicos e ambientais, sendo então essenciais para o planejamento e monitoramento das intervenções em saúde^7^. Mudanças no padrão epidemiológico brasileiro, caracterizado pelo aumento da prevalência de doenças crônicas não transmissíveis^11^, reafirmam a importância da realização de inquéritos específicos para o estudo desses agravos e de seus fatores de risco.

O Estudo de Riscos Cardiovasculares em Adolescentes (ERICA) foi desenvolvido com o objetivo de estimar a prevalência de síndrome metabólica e obesidade em adolescentes brasileiros e traçar o perfil de fatores de risco para doenças cardiovasculares, como níveis de lipídios, de pressão arterial e de marcadores inflamatórios e de resistência à insulina. Sua base de dados agrega diversos conjuntos de informações, questionário, medidas antropométricas, recordatório alimentar e parâmetros bioquímicos do sangue, que respondem aos diversos objetivos.

O percentual de participação em estudos epidemiológicos, independentemente do desenho de estudo, tem diminuído ao longo dos anos. Apesar disso, estudos que descrevam esse percentual de resposta e as características de participantes e não participantes são escassos^8^ ^,^ ^9^ ^,^ ^14^. Quanto maior o percentual de participação em um estudo, menor é a probabilidade de os resultados serem afetados por comprometimento da validade interna em função de viés de autoseleção^4^ ^,^ ^8^.

Este estudo teve como objetivo descrever o percentual de resposta e características de participantes e não-participantes no ERICA segundo subconjuntos de informações.

## MÉTODOS

O ERICA é um inquérito de base escolar de abrangência nacional. A amostra foi desenhada para representar adolescentes, de 12 a 17 anos de idade, que estudam em escolas públicas ou privadas, nos turnos da manhã ou da tarde, em municípios de mais 100 mil habitantes em todo o Brasil.

Trata-se de amostra de desenho complexo, com 32 estratos, compostos pelas capitais dos estados e pelos conjuntos formados pelos municípios de mais 100 mil habitantes dos interiores das macrorregiões brasileiras. Em cada estrato foram selecionadas escolas, com probabilidades proporcionais ao número de alunos matriculados nos sétimo, oitavo e nono anos do nível fundamental e nos primeiro, segundo e terceiro anos do ensino médio e inversamente proporcional à distância do município da escola para a capital do estado. Nessas escolas, três combinações de ano e turno (manhã e tarde) foram selecionadas. Em cada combinação foi selecionada uma turma e todos os seus alunos foram convidados a participar do estudo. No total foram selecionadas 1.251 escolas em 124 municípios, de um total de 273 municípios com mais de 100 mil habitantes. Os resultados do estudo têm representatividade nacional para os 32 estratos e para as cinco macrorregiões brasileiras. Os detalhes sobre o desenho amostral podem ser vistos em Vasconcellos et al.^15^

Foi feito contato inicial com a direção das escolas sorteadas e, em caso de recusa na participação, foi permitida a substituição da escola por outra do mesmo estrato, com probabilidade de inclusão mais próxima possível, da mesma natureza de administração, isto é, pública ou privada, mesma localização urbana ou rural e com a mesma seleção de combinações de ano e turno. As exigências dos comitês de ética em pesquisa (CEP) para obtenção de informações variaram de acordo com o estado. O termo de consentimento livre e esclarecido (TCLE) foi exigido para todos os estudantes que tiveram sangue coletado. Entretanto, para as demais informações (questionário, antropometria, aferição da pressão arterial e recordatório alimentar), foi exigido apenas o termo de assentimento do aluno, concordando em participar, exceto em quatro estados (Goiás, Bahia, Roraima e Mato Grosso do Sul) e em escolas estaduais de Minas Gerais, que exigiram o TCLE assinado por responsável. O protocolo do estudo está descrito detalhadamente em Bloch et al.^3^

A coleta de informações foi realizada de forma a garantir a privacidade de cada aluno, ocorrendo preferencialmente na seguinte ordem:

•Questionário do adolescente: abrangeu questões específicas para cada um dos 11 blocos temáticos (características sociodemográficas, trabalho e emprego, atividade física, comportamento alimentar, tabagismo, consumo de álcool, saúde reprodutiva, saúde bucal, morbidade referida, duração do sono e transtorno mental comum). Foi autopreenchido em coletor eletrônico de informações, *Personal Digital Assistant* (PDA), contando com cerca de 105 questões (variações do número de questões dependendo do sexo). O preenchimento do questionário não permitia que as questões não fossem respondidas ou “puladas”, ou seja, mesmo que o adolescente respondesse não ter determinado hábito, precisava responder às questões subsequentes sobre esse hábito. Essa estratégia visou avaliar a consistência das respostas e evitar que o aluno optasse por negar um hábito para não ter que detalhá-lo subsequentemente. Algumas questões tinham como opção: “Não sei; não lembro; não quero responder”^3^.

- Medidas antropométricas: foram aferidas por observadores treinados. O peso foi aferido por medida única em balança eletrônica Líder^®^ com capacidade de 200 quilogramas e variação de 50 gramas; a estatura foi obtida pela média das duas aferições realizadas sequencialmente em estadiômetro portátil e desmontável da marca Alturexata^®^, com resolução de milímetro e campo de uso de até 213 centímetros; a circunferência de cintura foi obtida pela média de duas mensurações sequenciais, utilizando fita antropométrica em fibra de vidro, com resolução de 1 milímetro e comprimento de 1,5 metro; e a circunferência de braço foi obtida em uma única medição, circundando a fita antropométrica na altura do ponto médio entre o acrômio e o olécrano^3^.
- Pressão arterial e batimentos cardíacos: avaliados em três medições consecutivas por observadores treinados, com intervalo de três minutos, em monitor digital (Omron^®^ 705-IT) validado para uso em adolescentes^12^. Foram aferidos no braço direito, com o aluno sentado e com os pés no chão, e com manguito de tamanho apropriado para o tamanho do braço. Foi utilizada apenas a média das duas últimas medidas^3^.
- Recordatório alimentar de 24 horas: o consumo alimentar das últimas 24 horas que antecederam à entrevista foi registrado, por entrevistadores treinados, em *notebooks* com programa específico desenvolvido para essa função.
- Coleta de sangue: foram coletadas amostras de sangue apenas nos alunos do turno da manhã que fizeram jejum de 12 horas. Foram dosados: colesterol total, colesterol-HDL, triglicerídeos, glicose, hemoglobina glicada e insulina. As coletas foram realizadas nas escolas em data agendada exclusivamente para esse fim^3^.

As informações sobre o número de alunos matriculados nas turmas selecionadas para participar do estudo foram obtidas nas escolas selecionadas. Foram confeccionados cadastros atualizados com os nomes e as datas de nascimento dos alunos que efetivamente frequentavam as turmas selecionadas. A informação sobre frequência dos alunos cadastrados era obtida nas turmas e com professores e diretores.

A estratégia geral de coleta foi coordenada pela equipe central do projeto, porém, em cada estado, havia uma coordenação local responsável por todos os aspectos da logística, pelo recrutamento e acompanhamento de supervisores, treinados pela coordenação central, e por todas as etapas do processo de obtenção de informações, que foi feita nas escolas por pesquisadores de campo contratados e treinados^3^.

No questionário autopreenchido pelos estudantes também eram registrados a idade em anos completos e o sexo. As informações sobre data de nascimento e idade foram utilizadas para identificar os adolescentes elegíveis para o estudo, já que alunos com idades inferiores a 12 anos e superiores a 17 anos poderiam estar matriculados nas turmas selecionadas. A estratégia de convidar todos os estudantes dos anos elegíveis objetivou facilitar a logística da coleta, incluir o maior número de alunos das idades-alvo, de 12 a 17 anos e não constranger alunos com idade fora da faixa elegível excluindo-os nessa etapa. Alunos da faixa etária elegível cursando anos inferiores ao sétimo ano não foram incluídos. Foram excluídos para análise os adolescentes que estivessem fora da faixa etária de 12 a 17 anos, as adolescentes grávidas e aqueles com deficiências físicas, temporárias ou permanentes, que não permitissem a aferição de medidas antropométricas com os instrumentos utilizados na pesquisa.

Foram construídos subconjuntos de adolescentes em função do conjunto de informações obtidas a fim de viabilizar o cálculo dos pesos amostrais. Os critérios de inclusão e de exclusão dos participantes do estudo elegíveis para constituição dos conjuntos completos de informações foram:

- Questionário: incluídos quando todas as questões compreendidas nos 11 blocos temáticos do questionário do aluno foram respondidas.
- Antropometria: incluídos quando pelo menos medidas de peso e estatura foram registradas.
- Pressão arterial: excluídas as medidas consideradas inválidas (pressão arterial sistólica igual ou menor que a pressão arterial diastólica).
- Recordatório alimentar de 24 horas: excluídos os com consumo energético inferior a 100 kcal.
- Sangue: incluído os com resultado de pelo menos um exame bioquímico dentre os seis previstos.

Assim, um determinado indivíduo pode ter preenchido o questionário, mas não respondeu ao recordatório. Dessa forma, os pesos amostrais foram calculados para cada combinação desses subconjuntos definidos, de forma a obter estimativas não enviesadas da população do estudo. Isso resultou em 11 subconjuntos finais: 1) questionário; 2) questionário e antropometria; 3) questionário e recordatório; 4) questionário, antropometria e pressão arterial; 5) questionário, antropometria e recordatório; 6) questionário, antropometria, pressão arterial e recordatório; 7) questionário e sangue; 8) questionário, antropometria e sangue; 9) questionário, antropometria, pressão arterial e sangue; 10) questionário, antropometria, recordatório e sangue; 11) questionário, antropometria, pressão arterial, recordatório e sangue (Figura).

**Figura:** Fluxograma dos adolescentes elegíveis e completude da amostra em relação aos blocos de informações e subconjuntos de informações. ERICA, Brasil, 2013-2014. (Continua) ERICA: Estudo de Riscos Cardiovasculares em Adolescentes; PA: pressão arterial

^a^ Menores de 12 anos e maiores de 17 anos de idade.

^b^ Entre os 102.327 elegíveis, 23,7% (24.284) não responderam a nenhum bloco de informação: questionário, antropometria, pressão arterial e recordatório de 24 horas.

^c^ Entre os 72.508 elegíveis do turno da manhã, 22,3% (16.131) não responderam a nenhum bloco de informação: questionário, antropometria, pressão arterial e recordatório de 24 horas.

^d^ Entre os 29.819 elegíveis do turno da tarde, 27,3% (8.153) não responderam a nenhum bloco de informação: questionário, antropometria, pressão arterial e recordatório de 24 horas.

Foram calculados percentuais de respostas efetivos, isto é, número de participantes com informações completas de acordo com o subconjunto de informações avaliado dividido pelo total de elegíveis (adolescentes da faixa etária elegível cadastrados nas turmas), segundo as macrorregiões, o sexo, a idade (categorizada como 12 a 14 e 15 a 17 anos), e tipo de escola (pública ou privada). Também, foram calculados os percentuais de escolas substitutas das selecionadas na amostra original, de acordo com o tipo de escola nas macrorregiões.

Os participantes e não-participantes foram comparados segundo sexo, idade e média de índice de massa corporal (IMC; kg/m^2^). Informações sobre sexo e idade dos não-participantes foram obtidas no cadastro da escola. O IMC foi obtido daqueles que participaram exclusivamente da etapa da antropometria, e daqueles que referiram peso e altura, mas que se recusaram a fornecer as demais informações do estudo.

Os não-participantes eram constituídos por: ausentes, alunos que não compareceram à escola no dia agendado para a coleta de informações do estudo; perdas parciais, aqueles que responderam parcialmente ao questionário, com < 100 kcal no R24h ou que não tiveram peso ou estatura aferidos; perdas de logística, aqueles que participaram em alguma etapa do estudo, porém suas informações não foram transferidas para o servidor central de dados em função de problemas operacionais; e recusa, aqueles que, embora presentes, não quiseram participar de alguma etapa.

## RESULTADOS

A coleta iniciou-se em uma escola pública no município do Rio de Janeiro, na região Sudeste, em 18 de março de 2013, e foi concluída em uma escola pública de Boa Vista, em Roraima, na região Norte, em 10 de novembro de 2014.

Dentre os 102.327 alunos cadastrados nas turmas selecionadas, 78.004 (76,2%) participaram de alguma etapa de coleta de dados do estudo. Na amostra total, a maior participação foi obtida para o subconjunto de informações do questionário, com percentuais um pouco menores ao se adicionarem medidas antropométricas ou do recordatório (Figura, A). Esses percentuais referem-se às informações obtidas em escolares das turmas da manhã e da tarde. A participação foi menor ao se incluir o exame de sangue, considerando-se apenas os estudantes do turno da manhã (Figura, B). De um modo geral, essas mesmas relações foram observadas de acordo com macrorregião, sexo, faixa etária e tipo de escola (Tabela 1).

**Tabela 1:** Percentual de resposta por sexo, idade e macrorregião. ERICA, Brasil, 2013-2014.

Variável sociodemográfica	Número de alunos		Questionário (%)			
	
	Total	Turno da manhã	A	B	C	D
Brasil	102.327	72.508	72,9	71,9	70,3	52,5
Sexo						
Feminino	54.403	38.632	75,8	75,0	73,4	59,1
Masculino	47.924	33.876	69,6	68,5	66,9	45,0
Faixa etária						
12-14	44.014	30.369	77,6	76,7	75,0	57,4
15-17	58.313	42.139	69,4	68,3	66,8	49,0
Norte	20.651	14.155	73,0	72,6	70,3	51,7
Sexo						
Feminino	10.924	7.539	75,2	74,8	72,6	57,2
Masculino	9.727	6.616	70,5	70,1	67,7	45,5
Faixa etária						
12-14	8.896	6.010	77,6	77,3	74,9	57,7
15-17	11.755	8.145	69,5	69,1	66,9	47,3
Nordeste	31.754	22.211	73,0	72,0	70,4	53,2
Sexo						
Feminino	17.063	11.914	75,4	74,6	72,9	59,9
Masculino	14.691	10.297	70,2	68,9	67,4	45,5
Faixa etária						
12-14	13.572	9.734	77,4	76,6	74,9	57,1
15-17	18.182	12.477	69,6	68,6	66,9	50,2
Centro-Oeste	14.238	10.281	68,3	67,5	66,0	53,6
Sexo						
Feminino	7.666	5.513	73,4	72,7	71,1	61,2
Masculino	6.572	4.768	62,3	61,3	60,0	44,9
Faixa etária						
12-14	6.142	4.336	73,5	72,6	71,6	60,7
15-17	8.096	5.945	64,4	63,5	61,8	48,5
Sudeste	23.911	17.506	71,4	70,4	69,2	49,4
Sexo						
Feminino	12.640	9.337	74,8	74,1	72,8	56,5
Masculino	11.271	8.169	67,6	66,4	65,2	41,3
Faixa etária						
12-14	10.260	6.907	77,1	76,3	74,8	53,8
15-17	13.651	10.599	67,2	66,0	65,0	46,6
Sul	11.773	8.355	81,0	79,2	77,8	56,9
Sexo						
Feminino	6.110	4.329	82,9	81,2	80,0	63,0
Masculino	5.663	4.026	79,0	77,0	75,5	50,4
Faixa etária						
12-14	5.144	3.382	83,7	82,1	80,2	60,9
15-17	6.629	4.973	79,0	76,9	75,9	54,2

A: Questionário Isolado; B: + antropometria; C: + recordatório; D: + sangue (turno da manhã).

Os maiores percentuais de resposta dos subconjuntos de informações que combinam questionário isolado com antropometria ou com recordatório foram obtidas na região Sul, especialmente na faixa etária mais velha, de 15 a 17 anos, em ambos os sexos (Tabela 1). Para a combinação das informações do questionário e do sangue, a maior participação ocorreu nos mais jovens, de 12 a 14 anos, do sexo feminino na região Centro-Oeste (Tabela 2).

**Tabela 2:** Cobertura de informações por sexo, idade e macrorregião. ERICA, Brasil, 2013-2014.

Variável sociodemográfica	Número de alunos		Questionário (%)			
	
	Total	Turno da manhã	A	B	C	D
Brasil						
Feminino						
12-14	23.303	16.094	80,3	79,6	78,0	64,0
15-17	31.100	22.538	72,4	71,5	69,9	55,6
Masculino						
12-14	20.711	14.275	74,5	73,5	71,7	50,0
15-17	27.213	19.601	65,9	64,7	63,2	41,4
Norte						
Feminino						
12-14	4.722	3.196	79,2	78,9	76,8	62,4
15-17	6.202	4.343	72,1	71,7	69,5	53,3
Masculino						
12-14	4.174	2.814	75,9	75,6	72,8	52,4
15-17	5.553	3.802	66,5	66,1	63,9	40,5
Nordeste						
Feminino						
12-14	7.290	5.217	80,3	79,7	77,7	64,5
15-17	9.773	6.697	71,7	70,9	69,3	56,3
Masculino						
12-14	6.282	4.517	74,1	72,9	71,7	48,6
15-17	8.409	5.780	67,2	65,9	64,2	43,1
Centro-Oeste						
Feminino						
12-14	3.244	2.291	78,5	77,8	77,0	68,0
15-17	4.422	3.222	69,7	69,0	66,8	56,4
Masculino						
12-14	2.898	2.045	67,8	66,8	65,6	52,5
15-17	3.674	2.723	58,0	57,0	55,7	39,1
Sudeste						
Feminino						
12-14	5.419	3.653	79,8	79,2	77,7	60,4
15-17	7.221	5.684	71,2	70,2	69,1	54,0
Masculino						
12-14	4.841	3.254	74,1	73,0	71,5	46,4
15-17	6.430	4.915	62,7	61,4	60,4	38,0
Sul						
Feminino						
12-14	2.628	1.737	85,5	84,0	82,8	67,5
1@@5-17	3.482	2.592	81,0	79,0	77,8	60,1
Masculino						
12-14	2.516	1.645	81,7	80,1	77,6	54,0
15-17	3.147	2.381	76,9	74,5	73,8	47,9

A: Questionário Isolado; B: + antropometria; C: + recordatório; D: + sangue (turno da manhã).

Para a maioria das combinações, a participação no estudo foi maior nas escolas privadas do que nas públicas. Porém, na região Sul, as diferenças foram menores e a participação dos adolescentes de 12 a 14 anos foi maior nas escolas públicas em todas as combinações de subconjuntos de informações; em relação ao subconjunto com sangue, essa maior participação nas escolas públicas foi observada nas duas faixas etárias e em ambos os sexos (Tabela 3).

**Tabela 3:** Percentual de resposta por sexo, idade, macrorregião e tipo de escola. ERICA, Brasil, 2013-2014.

Variável sociodemográfica	Escolas públicas						Escolas privadas					
	
	Número de alunos		Questionário (%)				Número de alunos		Questionário (%)			
			
	Total	Turno da manhã	A	B	C	D	Total	Turno da manhã	A	B	C	D
Brasil	81.970	54.361	71,6	70,7	68,9	51,8	20.357	18.147	78,0	76,9	76,0	54,6
Sexo												
Feminino	43.691	29.024	74,6	73,9	72,0	58,6	10.712	9.608	80,5	79,4	78,8	60,5
Masculino	38.279	25.337	68,2	67,1	65,4	44,0	9.645	8.539	75,3	74,1	73,0	47,9
Faixa etária												
12-14	34.312	22.031	76,6	75,8	73,9	56,8	9.702	8.338	81,0	80,1	79,0	59,0
15-17	47.658	32.330	68,0	67,1	65,3	48,4	10.655	9.809	75,3	73,9	73,3	50,8
Norte	18.001	11.657	72,8	72,5	70,0	51,6	2.650	2.498	74,2	73,2	72,5	52,5
Sexo												
Feminino	9.579	6.263	74,9	74,7	72,2	57,0	1.345	1.276	77,0	75,8	75,7	57,7
Masculino	8.422	5.394	70,4	70,1	67,5	45,2	1.305	1.222	71,4	70,6	69,2	47,1
Faixa etária												
12-14	7.927	5.185	77,4	77,2	74,5	57,7	969	825	79,5	78,7	78,4	57,9
15-17	10.074	6.472	69,2	68,9	66,5	46,7	1.681	1.673	71,2	70,1	69,1	49,9
Nordeste	23.704	14.741	71,0	70,1	68,1	52,2	8.050	7.470	78,6	77,6	76,8	55,3
Sexo												
Feminino	12.774	7.913	73,3	72,6	70,6	59,1	4.289	4.001	81,5	80,6	79,6	61,3
Masculino	10.930	6.828	68,4	67,1	65,3	44,1	3.761	3.469	75,3	74,2	73,7	48,3
Faixa etária												
12-14	9.199	5.757	75,9	75,0	73,0	56,3	4.373	3.977	80,7	79,8	79,0	58,3
15-17	14.505	8.984	68,0	67,0	65,1	49,6	3.677	3.493	76,0	75,0	74,2	51,8
Centro-Oeste	11.194	7.474	66,8	65,9	64,2	52,2	3.044	2.807	73,8	73,2	72,6	57,5
Sexo												
Feminino	6.022	3.993	72,4	71,6	69,8	60,5	1.644	1.520	77,2	76,7	76,2	63,1
Masculino	5.172	3.481	60,3	59,2	57,8	42,6	1.400	1.287	69,8	69,1	68,4	50,9
Faixa etária												
12-14	4.640	2.965	71,8	70,8	69,7	58,8	1.502	1.371	78,6	78,2	77,5	64,8
15-17	6.554	4.509	63,3	62,4	60,3	47,8	1.542	1.436	69,1	68,3	67,8	50,6
Sudeste	19.780	14.042	69,7	68,7	67,3	48,5	4.131	3.464	79,8	78,6	78,2	53,3
Sexo												
Feminino	10.451	7.499	73,4	72,7	71,2	55,6	2.189	1.838	81,6	80,5	80,4	60,2
Masculino	9.329	6.543	65,5	64,3	63,0	40,3	1.942	1.626	77,8	76,5	75,7	45,5
Faixa etária												
12-14	8.445	5.445	75,6	74,8	73,1	52,4	1.815	1.462	84,0	83,0	82,8	59,0
15-17	11.335	8.597	65,3	64,2	63,1	46,0	2.316	2.002	76,6	75,1	74,7	49,2
Sul	9.291	6.447	80,7	78,9	77,8	58,3	2.482	1.908	82,4	80,2	77,8	52,4
Sexo												
Feminino	4.865	3.356	82,9	81,2	80,1	64,8	1.245	973	83,1	81,0	79,5	56,9
Masculino	4.426	3.091	78,3	76,3	75,3	51,2	1.237	935	81,7	79,4	76,0	47,7
Faixa etária												
12-14	4.101	2.679	84,0	82,5	81,5	62,9	1.043	703	82,3	80,5	75,5	53,5
15-17	5.190	3.768	78,1	76,0	74,9	55,0	1.439	1.205	82,5	79,9	79,4	51,8

A: Questionário Isolado; B: + antropometria; C: + recordatório; D: + sangue (turno da manhã).

Das 1.251 escolas selecionadas na amostra original, quatro foram perdidas: três em São Paulo e uma no Amapá, todas privadas, sem substituição. No final, 139 (11,1%) das escolas foram substituídas, sendo que entre as 1.012 escolas públicas e as 235 escolas privadas, essas substituições ocorreram, respectivamente, em 6,0% e 32,2% dos casos. Em todas as regiões, as substituições foram relativamente mais frequentes nas escolas privadas. A região Norte apresentou a menor frequência relativa de substituições nas escolas privadas, entretanto a região Sul apresentou os extremos, isto é, o menor percentual de substituições nas escolas públicas (3,8%) e o maior percentual nas privadas (48,4%). A cobertura do questionário nas escolas públicas que não foram substituídas foi de 71,7% e nas substitutas foi de 70,7%, enquanto que nas escolas privadas os percentuais correspondentes foram de 78,4% e 77,0% (Tabela 4).

**Tabela 4:** Percentual de resposta de questionários por escolas originalmente selecionadas ou substitutas por macrorregião. ERICA, Brasil, 2013-2014.

Macrorregião	Escolas públicas				Escolas privadas			
	
	Seleção original		Substitutas		Seleção original		Substitutas	
			
	n	%	n	%	n	%	n	%
Brasil	77.459	71,7	4511	70,7	14.400	78,4	5.957	77,0
Norte	17.028	72,8	973	72,8	2.334	74,1	316	75,3
Nordeste	22.336	70,9	1368	73,0	6.126	79,7	1.924	74,9
Centro-Oeste	10.773	66,9	421	64,8	2.246	76,3	798	66,8
Sudeste	18.267	69,7	1513	69,3	2.372	78,5	1.759	81,6
Sul	9.055	81,0	236	68,2	1.322	83,7	1.160	80,9

Quanto ao subconjunto de informações do questionário e antropometria, entre os 28.713 elegíveis não-participantes, a grande maioria (77,2%) estava ausente no dia da coleta de informações em ambas as etapas, 5.272 (19,0%) estavam presentes e se recusaram a participar, 109 (0,4%) foram perdas de logística e 1.154 (4,0%) foram perdas parciais (realizaram aferição de peso ou estatura ou ambos, mas não responderam o questionário; ou apenas responderam o questionário). O IMC foi obtido em 2.374 adolescentes não-participantes, dos quais, 2.214 (93,2%) eram recusas e autorreferiram seu peso e estatura, e 160 (6,8%) eram perdas parciais e tiveram as medidas de peso e estatura aferidas, apesar de não responderam ao questionário (Tabela 5).

**Tabela 5:** Percentuais e índice de massa corporal (IMC) de participantes e não participantes, segundo sexo e faixa etária por macrorregião, na amostra de elegíveis do ERICA, Brasil, 2013-2014.

Variável sociodemográfica	Participantes^a^				Não participantes					
	
	n	%	IMC	IC95%	n	%	n^b^	%	IMC	IC95%
Brasil	73.624	-	21,2	21,2-21,2	28.703	-	2.374	-	21,4	21,3-21,6
Sexo										
Feminino	40.803	55,4	21,3	21,3-21,4	13.600	47,4	945	39,8	21,4	21,2-21,7
Masculino	32.821	44,6	21,0	21,0-21,0	15.103	52,6	1.429	60,2	21,4	21,2-21,7
Faixa etária										
12-14	33.778	45,9	20,5	20,5-20,6	10.236	35,7	1.085	45,7	20,8	20,5-21,0
15-17	39.846	54,1	21,7	21,7-21,8	18.467	64,3	1.289	54,3	22,0	21,8-22,2
Norte	14.996	-	20,9	20,9-21,0	5.655	-	245	-	21,6	21,1-22,1
Sexo										
Feminino	8.174	54,5	21,0	20,9-21,1	2.750	48,6	97,0	39,6	21,1	20,4-21,8
Masculino	6.822	45,5	20,8	20,7-20,9	2.905	51,4	148	60,4	21,9	21,2-22,6
Faixa etária										
12-14	6.879	45,9	20,2	20,1-20,3	2.017	35,7	114	46,5	21,0	20,2-21,7
15-17	8.117	54,1	21,6	21,5-21,6	3.638	64,3	131	53,5	22,1	21,4-22,8
Nordeste	22.864	-	21,1	21,0-21,1	8.890	-	244	-	21,8	21,2-22,4
Sexo										
Feminino	12.737	55,7	21,2	21,1-21,2	4.326	48,7	98	40,2	22,2	21,1-23,2
Masculino	10.127	44,3	20,9	20,8-21,0	4.564	51,3	146	59,8	21,5	20,8-22,2
Faixa etária										
12-14	10.390	45,4	20,5	20,4-20,6	3.182	35,8	97	39,8	21,5	20,4-22,5
15-17	12.474	54,6	21,5	21,5-21,6	5.708	64,2	147	60,2	22,0	21,3-22,7
Centro-Oeste	9.604	-	21,1	21,0-21,2	4.634	-	718	-	20,8	20,5-21,0
Sexo										
Feminino	5.573	58,0	21,2	21,1-21,3	2.093	45,2	304	42,3	20,8	20,4-21,2
Masculino	4.031	42,0	20,9	20,8-21,0	2.541	54,8	414	57,7	20,8	20,4-21,1
Faixa etária										
12-14	4.459	46,4	20,4	20,3-20,5	1.683	36,3	367	51,1	20,3	19,9-20,6
15-17	5.145	53,6	21,7	21,6-21,8	2.951	63,7	351	48,9	21,3	20,9-21,7
Sudeste	16.841	-	21,3	21,3-21,4	7.070	-	602	-	21,6	21,2-21,9
Sexo										
Feminino	9.360	55,6	21,6	21,5-21,7	3.280	46,4	225	37,4	21,9	21,2-22,6
Masculino	7.481	44,4	21,0	20,9-21,1	3.790	53,6	377	62,6	21,4	21,0-21,8
Faixa etária										
12-14	7.827	46,5	20,7	20,6-20,8	2.433	34,4	266	44,2	20,8	20,3-21,4
15-17	9.014	53,5	21,9	21,9-22,0	4.637	65,6	336	55,8	22,2	21,7-22,7
Sul	9.319	-	21,8	21,7-21,8	2.454	-	565	-	22,0	21,6-22,3
Sexo										
Feminino	4.959	53,2	21,9	21,3-22,0	1.151	46,9	221	39,1	21,7	21,3-22,2
Masculino	4.360	46,8	21,6	21,4-21,7	1.303	53,1	344	60,9	22,1	21,7-22,6
Faixa etária										
12-14	4.223	45,3	21,1	21,0-21,2	921	37,5	241	42,7	21,2	20,7-21,7
15-17	5.096	54,7	22,3	22,2-22,4	1.533	62,5	324	57,3	22,5	22,1-22,9

^a^ Em relação ao subconjunto de informações dos elegíveis que realizaram o questionário e a antropometria.

^b^ Número de indivíduos com informações de antropometria auto-referidos entre os que recusaram a participação no estudo (n = 2.214) ou que participou da etapa da antropometria, mas que não respondeu ou não chegou a completar o questionário (n = 160).

A proporção de adolescentes do sexo masculino e daqueles com 15 a 17 anos foram maiores entre os não participantes quando comparados aos participantes do ERICA. Essa configuração foi similar em todas as macrorregiões do País. Na amostra total e nas macrorregiões, a média de IMC foi um pouco maior entre os não-participantes (Tabela 5).

## DISCUSSÃO

Ainda que a robustez do tamanho amostral do ERICA e o cuidadoso planejamento e execução do estudo^3^ reforce a significância dos seus achados, é importante entender a dimensão e as características da participação no estudo frente a possíveis vieses que comprometam a sua qualidade interna.

Cerca de 78 mil adolescentes elegíveis tiveram alguma informação coletada no estudo e houve variações nos percentuais de participação de acordo com o subconjunto de informações. A maior participação foi para o subconjunto de informações coletadas no questionário autopreenchível e a menor para o subconjunto do sangue. Além disso, foram observadas algumas diferenças regionais e por tipo de escola: as escolas públicas apresentaram menor percentual de resposta quando comparadas com as escolas particulares, com exceção no Sul, especialmente em relação à coleta de sangue.

A avaliação sobre a participação e não participação em estudos epidemiológicos e a descrição detalhada dessa não-resposta não é encontrada com frequência na literatura^4^ ^,^ ^8^. É de grande importância que as não-respostas sejam devidamente relatadas para que os resultados possam ser interpretados de forma criteriosa e que estratégias de redução da não-resposta possam ser desenvolvidas^4^ ^,^ ^10^.

Morton et al.^8^ selecionaram 355 estudos epidemiológicos publicados de janeiro de 2003 a abril de 2003 em 10 periódicos internacionais, e identificaram 86 estudos transversais, dos quais apenas 51,0% relatavam sobre a participação, que variou entre 28,0% a 100%.

Estudos nacionais de base populacional em adolescentes são escassos^1^ ^,^ ^5^ ^,^ ^6^ ^,^ ^13^. A Pesquisa Nacional de Saúde do Escolar (PeNSE) realizada em 2012 com estudantes do nono ano do ensino fundamental^5^ obteve um percentual de resposta de 83,0%, considerando apenas o subconjunto de informação do questionário. A diferença entre os percentuais de resposta do ERICA e da PeNSE pode ser explicada, em parte, pela amostra da PeNSE incluir adolescentes apenas de 13 a 15 anos, mais jovens do que os do ERICA. No ERICA, a cobertura em todos os subconjuntos de informações foi maior entre os adolescentes de 12 a 14 anos, sendo de 76,2% para o subconjunto do questionário nessa faixa etária. Outra diferença entre os inquéritos é a obrigatoriedade do TCLE para alguns estados no ERICA, nos quais foram observados os percentuais de cobertura mais baixos. As regiões Centro-Oeste e Sul foram as que apresentaram o menor e o maior menor percentual de cobertura, respectivamente, em ambos os inquéritos.

Quando comparado a inquéritos internacionais como o estudo *Healthy Lifestyle in Europe by Nutrition in Adolescence* (HELENA)^2^, inquérito escolar que foi conduzido em 10 cidades da Europa em adolescentes com idade de 12 a 17 anos, a cobertura do ERICA foi superior (67,0% *versus* 73,0%). No HELENA, na subamostra dos adolescentes que realizou coleta de sangue, a cobertura para essas informações foi superior ao encontrado no ERICA (68,0% *versus* 52,0%).

Os diferenciais de participação nos subconjuntos de informações podem ser explicados pelo grau de complexidade na logística necessária para a obtenção das informações. A necessidade de TCLE para procedimentos não invasivos pode ter impactado nas perdas, inclusive nos subconjuntos de informação que não o do sangue. No Sul do País não houve exigência de TCLE; no Sudeste, apenas a Secretaria Estadual de Educação de Minas Gerais exigiu; nas demais macrorregiões houve exigência somente nos estados de Roraima no Norte, na Bahia no Nordeste e em Goiás e Mato Grosso do Sul no Centro-Oeste. Nesses últimos quatro estados, a cobertura de informações no questionário foi inferior a 60,0%. Isso pode refletir o impacto da obrigatoriedade do TCLE, visto que os outros dois estados da região Centro-Oeste, nos quais o TCLE não foi exigido, apresentaram percentual de resposta nos questionários superior a 80,0%.

As diferenças observadas entre os percentuais de resposta de acordo com o tipo de escola não parecem ter ocorrido pela maior proporção de substituição nas escolas privadas, uma vez que os percentuais de resposta entre escolas originais da amostra e das escolas substitutas foram semelhantes de forma independente da natureza administrativa da escola. É possível especular que as diferenças de cobertura de escolas públicas e privadas estejam relacionadas com a falta às aulas. Na PeNSE 2012 foi observado que, entre os alunos das escolas públicas, 28,8% referiram ter faltado às aulas sem permissão dos pais nos últimos 30 dias, enquanto que entre os alunos das escolas privadas esse percentual foi de 14,4%. Outras possíveis explicações são a localização mais frequente de escolas públicas em áreas de conflito e a maior probabilidade de os alunos trabalharem, o que pode aumentar o absenteísmo. Em relação a esta última hipótese observou-se, na amostra total de elegíveis, que 6,2% dos entrevistados de escolas públicas tinham trabalho remunerado, comparado a apenas 2,8% daqueles de escolas privadas (dados não apresentados). A estimativa da prevalência de alunos de escolas públicas que referiram ter algum trabalho pode estar subestimada, pois foi obtida apenas entre os que estavam presentes no dia da coleta e responderam ao questionário.

Os adolescentes que não participantes do ERICA eram em sua maioria do sexo masculino e da faixa etária de 15 a 17 anos. Além disso, apresentaram valores médios de IMC ligeiramente superiores aos dos adolescentes participantes do estudo. No entanto, dados de IMC foram estimados somente em 8,5% dos não participantes, portanto, essa estimativa pode não ser representativa do total de não participantes. No ERICA, foram implementados procedimentos de análise que, além de considerarem amostra complexa, usaram pesos calibrados que corrigem as estimativas de prevalência de acordo com a distribuição de idade e sexo em cada estrato da amostra, estimada com informações obtidas em censos populacionais^15^. Mais ainda, essas estimativas pontuais consideram que os participantes do ERICA representam os que não participaram. As possíveis perdas de precisão nas estimativas estão relacionadas mais com o nível de conglomeração, pela opção de incluir todos os alunos das turmas selecionadas, do que com o número total de observações, que se aproximou do esperado.

O ERICA adotou algumas estratégias visando estimular a participação dos alunos, tais como: elaboração de material explicativo sobre o estudo, confeccionado por renomado cartunista infantil de forma a atrair a atenção dos jovens – cartilha, cartaz e identidade visual da página do estudo; distribuição de kit-lanche para aqueles que fizeram jejum para coleta de sangue; e divulgação do estudo em página própria (www.erica.ufrj.br), com um canal de contato (fale conosco), e em mídia televisiva e impressa local no início da coleta em cada estado. Essas estratégias aumentaram o custo do estudo, mas são fundamentais para a redução da não-participação em estudos epidemiológicos. Da mesma forma, estratégias para alcançar os indivíduos não-participantes e obter deles algumas informações que possam caracterizá-los e ajudar a entender a resistência em participar são importantes e exigem um grande esforço logístico, de difícil implementação e alto custo em um grande estudo populacional^4^.

O ERICA é o primeiro inquérito nacional de base escolar que obteve informações abrangentes do estado nutricional, pressão arterial e parâmetros bioquímicos do sangue de adolescentes. A proporção de não-resposta, inferior a um terço, e com as características descritas, dificilmente causará impacto significativo nas estimativas do estudo. O presente estudo fornece informações úteis para futuros inquéritos em adolescentes que poderão aprimorar as estratégias de coleta de informações visando a reduzir a não-resposta.
